# Myocardial fibrosis associates with lupus anticoagulant in patients with systemic lupus erythematosus

**DOI:** 10.1007/s10554-023-02970-3

**Published:** 2023-10-09

**Authors:** Katrine A. Myhr, Amanda H. Zinglersen, Redi Pecini, Søren Jacobsen

**Affiliations:** 1 Department of Cardiology, Rigshospitalet, Inge Lehmanns Vej 7, 2100 Copenhagen, Denmark; 2 Copenhagen Research Center for Autoimmune Connective Tissue Diseases, Center for Rheumatology and Spine Diseases, Rigshospitalet, Juliane Maries Vej 10, 2100 Copenhagen, Denmark

**Keywords:** Systemic lupus erythematosus, Myocardial fibrosis, Cardiac magnetic resonance, T1 mapping, Lupus anticoagulant, Pathophysiology

## Abstract

Systemic lupus erythematosus (SLE) is a chronic autoimmune disease that carries increased risk of cardiovascular disease; however, the underlying pathophysiological mechanisms remain poorly understood. We aimed to investigate the prevalence and degree of myocardial fibrosis in SLE patients and associated disease characteristics. Forty-nine SLE patients (89% female, mean age 52 ± 13 years, median disease duration 19 (11–25) years) and 79 sex-and age-matched healthy controls were included. CMR with T1 mapping was performed on SLE patients and healthy controls. Fifty-one SLE patients received gadolinium contrast for the evaluation of late gadolinium enhancement (LGE) and extra cellular volume (ECV). Multiple linear regression analyses were performed to investigate the association between markers of myocardial fibrosis on CMR (LGE, T1, ECV) and SLE-related variables [clinical disease activity, lupus nephritis, chronic kidney disease, anti-cardiolipin and/or anti-beta-2 glycoprotein I antibodies, and lupus anticoagulant (LAC)] with adjustment for traditional risk factors. T1 values were elevated in SLE patients compared to healthy controls (1031 ± 36 ms vs. 1019 ± 25 ms, p = 0.01). LGE was present in 20% of SLE patients who received gadolinium contrast. On multivariable analysis, LAC was associated with LGE in SLE patients (β = 3.87, p = 0.02). Neither T1 nor ECV associated with SLE disease characteristics; however, there was a trend towards an association between LAC and T1 (β = 16.9, p = 0.08). SLE patients displayed signs of myocardial fibrosis on CMR that were associated with the presence of LAC. These findings support the pathophysiological understanding of LAC as a mediator of microvascular and subsequent myocardial dysfunction.

**Graphical abstract:**

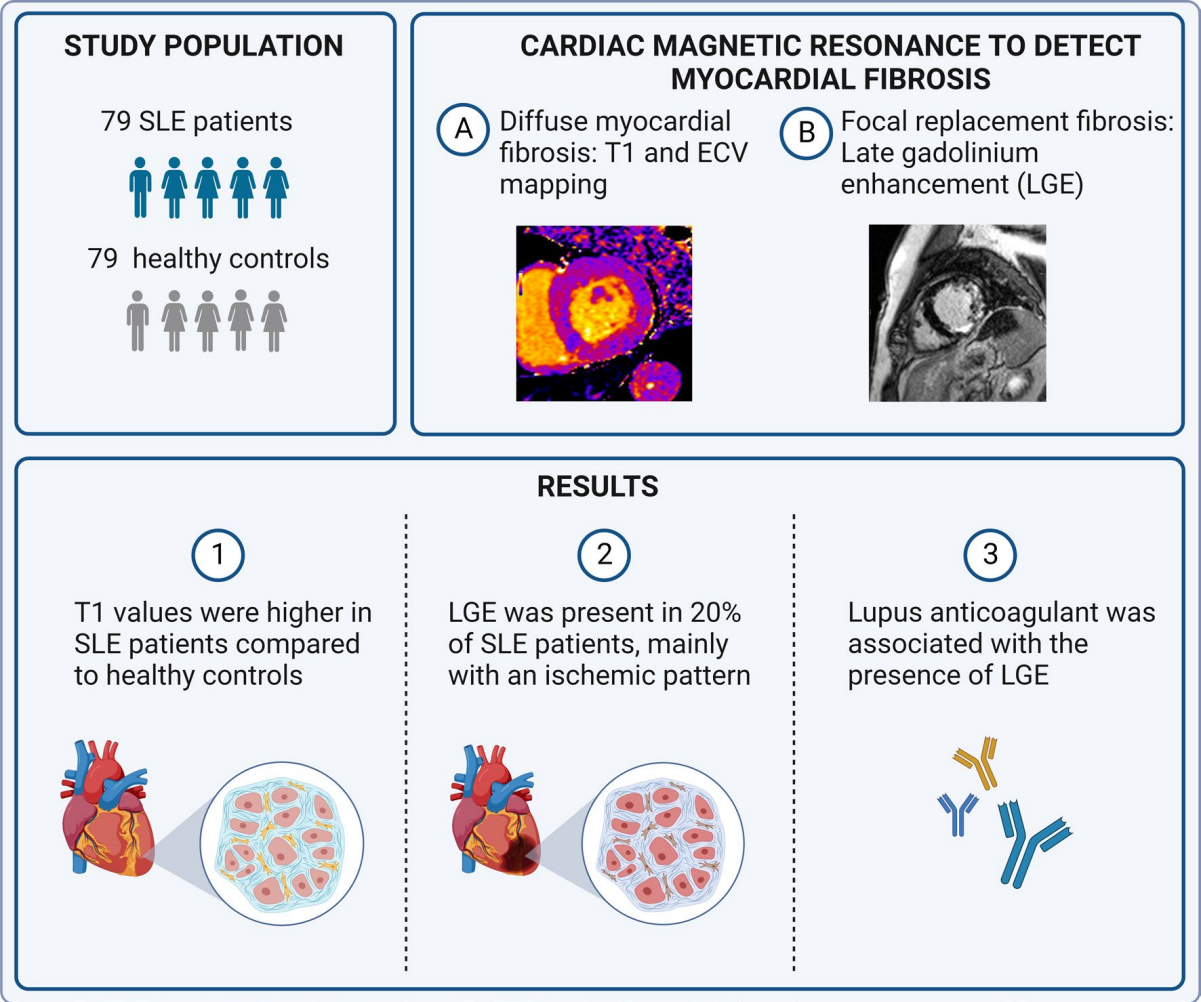

## Introduction

Patients with systemic lupus erythematosus (SLE) are at increased risk of cardiovascular disease, including myocardial infarction [1, 2], valvular heart disease [3], myocarditis [4], pericarditis [5], and heart failure [6, 7]. Even though SLE patients have increased prevalence of traditional cardiovascular risk factors [8], other SLE-related risk factors are equally believed to contribute such as disease activity, medications, nephritis, antiphospholipid antibodies (aPL), and lupus anticoagulant (LAC) [9]. However, the underlying mechanisms needs further exploration.

Previous studies have reported of an increased prevalence of myocardial fibrosis in SLE [10–14]. Myocardial fibrosis is involved in myocardial remodeling and associated with heart failure [15]. The question therefore seems, if myocardial fibrosis is an underlying cause of cardiac dysfunction observed in SLE patients, including cardiac remodeling, diastolic and systolic dysfunction [16, 17], and which disease factors promote the development.

Two types of myocardial fibrosis exist: replacement fibrosis and diffuse fibrosis. Replacement fibrosis is irreversible and usually develops due to myocyte necrosis (e.g. following ischemia [18]) but is also present in other conditions such as myocarditis [19], non-ischemic dilated cardiomyopathy [20], aortic valve stenosis [21], hypertrophic cardiomyopathy [22], and sarcoidosis [23]. Diffuse fibrosis can precede replacement fibrosis and is regarded as partly reversible. It can be reactive (e.g., due to age and hypertension [24]) or infiltrative (e.g., due to amyloidosis [25]).

Myocardial fibrosis can be estimated by cardiac magnetic resonance (CMR). Replacement fibrosis is visualized by late gadolinium enhancement (LGE) following administration of a gadolinium contrast agent. LGE imaging is part of routine CMR and has been validated against histology in animal models and humans [26–28]. The appearance of LGE requires a heterogenous fibrotic distribution, and LGE imaging is therefore unable to detect diffuse myocardial fibrosis. However, T1 and extracellular volume (ECV) mapping are novel semi-automated methods to quantify diffuse myocardial fibrosis. T1 and ECV values correlate with fibrosis on histology [29–31], but are also elevated in the presence of edema [32].

We aim to investigate the prevalence of myocardial fibrosis detected by CMR in a population of SLE patients and further associate CMR markers of myocardial fibrosis with biochemical and/or clinical SLE-related risk factors. These include LAC that by our group has been found to be associated with myocardial remodeling and impaired diastolic function [17]. With this, we hope to expand the pathophysiological understanding of cardiac dysfunction in SLE, which seems crucial for better risk stratification and individual follow-up.

## Methods

### Study population

A total of 79 patients with an established diagnosis of SLE according to the 1997 American College of Rheumatology Revised criteria were included from October 2018 through March 2019 from our tertiary referral center for systemic autoimmune diseases at Copenhagen University Hospital, Rigshospitalet, Denmark. Patients were recruited as previously described [33]. Patients were 18 years of age or older. Pregnancy and ischemic heart disease [i.e., previous primary coronary intervention (PCI) and/or coronary artery bypass grafting (CABG)] were exclusion criteria.

Physical examination, blood samples, and information regarding current medication, previous cardiovascular events, disease activity by the Systemic Lupus erythematosus Disease Activity Index 2000 (SLEDAI-2 K), lupus nephritis (LN), chronic kidney disease stage (CKD), anti-cardiolipin antibodies (aCL), anti-beta2 glycoprotein antibodies (aB2GP), lupus anticoagulant (LAC), organ damage, and traditional cardiovascular risk factors were performed and obtained as also previously described by our group [17].

Further, 79 healthy sex- and age-matched controls were included. The inclusion criterion was age 18–89 years. Exclusion criteria were pregnancy or other contraindications for CMR (e.g., claustrophobia or metallic implants) as well as history of cardiovascular disease (e.g., coronary artery disease, hypertension, and hypercholesterolemia) or other chronic diseases such as chronic obstructive lung disease, chronic kidney disease, and rheumatological disease.

### Cardiac magnetic resonance

All patients and healthy controls underwent a CMR examination on a 1.5 T Siemens Magnetom Area Scanner. Steady state free precession images were obtained during breath-hold for the analysis of cardiac morphology and function. Typical cine image parameters were as follows: field-of-view (FOV) 340 mm × 276.4 mm^2^, in-plane resolution 1.8 × 1.8 × 8 mm^3^, TR/TE = 42.4/1.11 ms, and flip angle 40°.

Three left ventricular (LV) long-axis planes (two-, three-, and four-chamber views) were acquired as well as a short-axis stack covering both ventricles without gaps.

Late gadolinium enhancement (LGE) imaging was performed only on SLE patients with an estimated eGFR ≥ 45 mL/min/1.73 m^2^. Gadobutrol (Gadovist, Bayer, Germany) was administered into a peripheral vein at a dose of 0.15 mg/kg. A TI-scout was performed to determine the appropriate TI-time for nulling of the myocardium. LGE images were acquired 12 min following contrast administration and included a full short-axis stack and three long-axis planes of the LV. The TI-time was continuously adjusted during acquisition. In case of LGE, an orthogonal image was acquired to ensure detectability in two planes. Typical LGE image parameters were as follows: FOV 340 × 277.8 m^2^, in-plane resolution 1.6 × 1.6 × 8 mm^3^, TR/TE = 700/3.09, and flip angle 25°.

Native T1 mapping was performed using a Look-Locker inversion recovery (MOLLI) sequence following a 5(3)3 protocol. Three short-axis subsets were acquired at the base, mid and apical part of the LV, respectively. T1 mapping images were repeated 10 min post-contrast administration for the generation of ECV mapping images. Typical T1 mapping image parameters were as follows: FOV 360 × 306.7 mm^2^, in-plane resolution 1.4 × 1.4 × 8 mm^3^, TR/TE = 280.6/1.12, and flip angle 35°.

Analysis of CMR images was performed using a commercially available software (Circle CVI 42^®^, Calgary, Canada). LV and right ventricular (RV) volumes were quantified using an AI-function automatically delineating the endo- and epicardium in all slices and phases. A visual validation was subsequently performed, and corrections were performed when necessary. LV and RV end-diastole and end-systole were defined as the phase with the largest and smallest volume, respectively.

Identification and location of LGE was verified by two separate observers in all patients. LGE was characterized in accordance with the Society for Cardiovascular Magnetic Resonance (SCMR) guidelines [34] as either (i) subendocardial or transmural or (ii) mid- and/or epicardial, representing an ischemic or non-ischemic injury, respectively. LGE was quantified by a semi-automated quantification method using either ± 5 SD in cases with an ischemic pattern and ± 3SD in cases with a non-ischemic pattern. LGE was expressed as % volume.

T1 mapping analyses were performed on motion-corrected images, and image quality was evaluated for significant artefacts such as motion and banding artefacts. T1 mapping images with significant artefacts were excluded for further analyses. The endo- and epicardial contours were drawn using an AI-function and corrected if tracings were visually incorrect. A 20% endo- and epicardial offset was chosen to reduce partial-voluming artefacts. A global T1 value was subsequently calculated. ECV maps were computed from pre- and post-contrast T1 images. Hematocrit was measured on the same day as the CMR examination and used in calculations of the ECV using the following formula: ${\text{ECV}}_{{{\text{CMR}}}} ~ = ~\left( {{\text{1}} - {\text{hematocrit}}} \right)~ \times ~\left( {\Delta \left( {{\text{1}}/{\text{T1}}_{{{\text{myo}}}} } \right)/\Delta \left( {{\text{1}}/{\text{T1}}_{{{\text{blood}}}} } \right)} \right)$.

### Echocardiography

A standard echocardiographic examination was performed on all patients using a Vivid E95 ultrasound scanner (GE Healthcare Vingmed ultrasound AS, Horten, Norway) and a M5Sc-D Matrix phased array transducer (1.5–4.6 MHz). Performance and analysis of echocardiographic examinations were as previously described [17] and included evaluation of cardiac chamber size, left ventricular ejection fraction (LVEF), and diastolic function.

### Statistical analysis

**Fig. 1:**
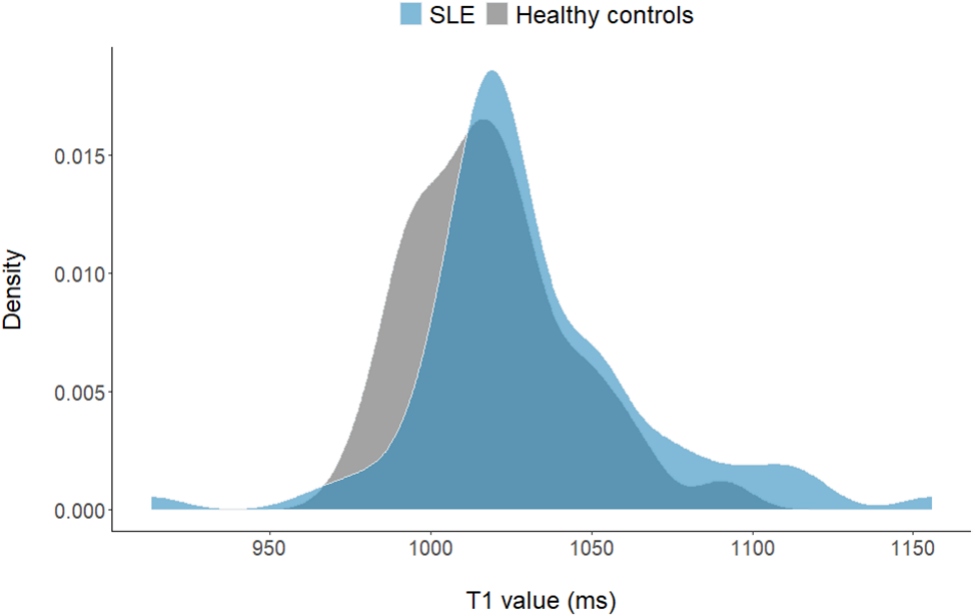
Density plot of T1 values in SLE patients (blue) compared with healthy controls (grey)

**Fig. 2:**
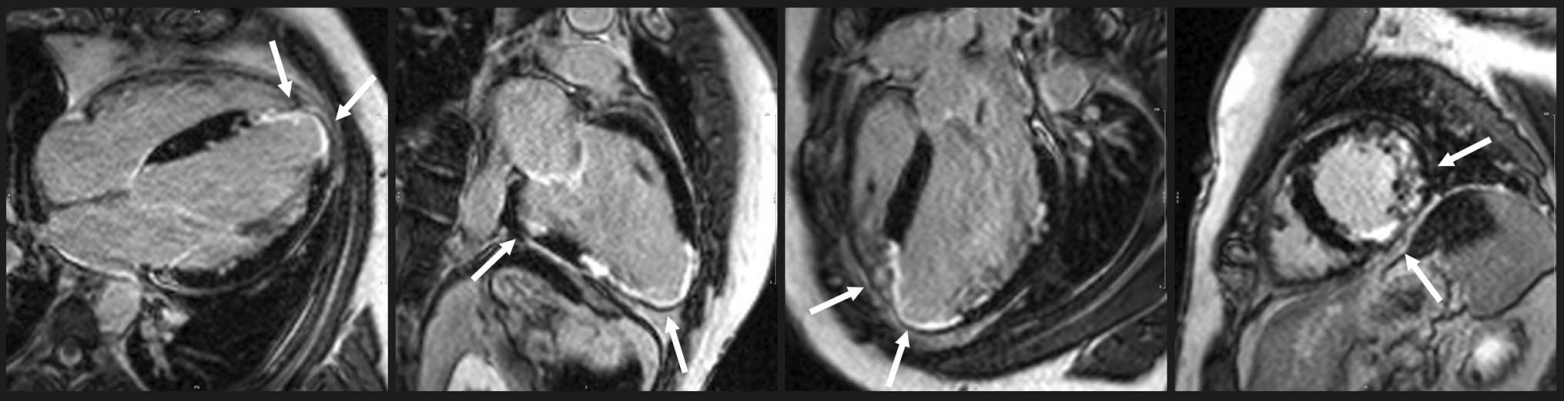
Late gadolinium enhancement in 35-year-old male patient with systemic lupus erythematosus, antiphospholipid syndrome, and presence of lupus anticoagulant but no history of heart disease. Cardiac magnetic resonance revealed systolic heart failure (left ventricular ejection fraction 35%) and late gadolinium enhancement (white arrows) in all three coronary territories. Coronary angiogram by computed tomography was normal, and the findings were interpreted as resulting from multiple micro emboli and/or thrombi

Continuous, normally distributed values are expressed as mean ± standard deviation and non-normally distributed values as median and quartiles. P-values ≤ 0.05 were considered statistically significant. Welch’s *t*-test was used for comparing normally-distributed data and the Wilcoxon singed-rank test for non-normally distributed data. Correlation between T1 and ECV values was assessed by the Pearson correlation coefficient. Multiple linear and logistic regression analyses were performed to investigate the associations between markers of myocardial fibrosis on CMR (LGE, T1 and ECV) and clinical and biochemical SLE-characteristics (SLEDAI-2 K, LN, CKD, IgM aCL and/or aB2GPI, IgG aCL and/or aB2GPI, and LAC). Further, linear and logistic regressions analyses were performed for the association between markers of myocardial fibrosis on CMR (LGE, T1 and ECV) and biventricular size and function on CMR and echocardiography. All analyses were adjusted for sex, age, hypertension, hypercholesterolemia, and smoking. Non-normally distributed variables were log-transformed to normal distribution before regression analyses. All statistical analyses and Fig. 1 were performed and created using R Statistical Software version 1.2.5001 (*R* Foundation for *Statistical* Computing, Vienna, Austria). The graphical abstract and Fig. 2 were created with BioRender.com.

## Results

### Study population

A total of 70 female and 9 male SLE patients with a mean age of 52 ± 13 years and a disease duration of 19 (11–25) years were included. Demographic and clinical baseline characteristics are presented in Table 1. Further, 79 healthy sex- and age-matched controls were included (89% females, age 49 ± 15 years).

**Table 1:** Demographic and clinical characteristics of 79 patients with systemic lupus erythematosus

Parameters	Value
Female, n (%)	70 (89)
Age, years	52 ± 13
BMI, kg/m^2^	25 ± 5
Blood pressure, mmHg	124 ± 16/72 ± 11
Arterial hypertension^a^, n (%)	42 (53)
Hypercholesterolemia^b^, n (%)	38 (48)
Smoking ever, n (%)	49 (62)
Diabetes, n (%)	4 (5)
Chronic kidney disease stages^c^, n (%)	
CKD 1	34 (43)
CKD 2	10 (13)
CKD 3	35 (44)
Pulmonary hypertension, n (%)	2 (3)
Valve surgery, n (%)	3 (4)
Disease duration, years	19 (11–25)
SLEDAI-2 K	3 (2–4)
SLICC damage index	2 (1–4)
Lupus nephritis^d^, n (%)	46 (58)
Antiphospholipid antibodies (aPL), n (%)	
IgM aCL and/or aB2GPI	15 (19)
IgG aCL and/or aB2GPI	18 (23)
LAC	22 (28)
Medication, n (%)	
Antihypertensives	37 (47%)
Corticosteroids	22 (28%)
Antimalarials	43 (54%)
Other immunosuppressants^e^	42 (53%)

V Values are mean ± standard deviation or median (quartiles)

*aB2GPI* anti-beta-2-glycoprotein I antibodies, *aCL* anti-cardiolipin antibodies, *BMI* Body Mass Index, *CKD* chronic kidney disease stage, *LAC* lupus anticoagulant, *SLE* systemic lupus erythematosus

^a^Resting systolic blood pressure > 140 mmHg or a resting diastolic blood pressure > 90 mmHg, and/or use of antihypertensive medication (β-blockers, diuretics, calcium inhibitors, angiotensin-converting enzyme inhibitors and angiotensin receptor blockers)

^b^Statin therapy and/or total cholesterol ≥ 6.2 mmol/L, low-density lipoprotein ≥ 4.1 mmol/L, triglycerides ≥ 2.3 mmol/L, or high-density lipoprotein < 1.0 mmol/L

^c^CKD 1: eGFR > 90 mL/min/1.73 m^2^; CKD 2: eGFR 60–89 mL/min/1.73 m^2^; CKD 3–5: eGFR 0–59 mL/min/1.73 m^2^

^d^Presence of persistent proteinuria according to the SLE classification criteria

^e^Azathioprine, cyclophosphamide, mycophenolate mofetil, cyclosporine, rituximab, and methotrexate

### Clinical imaging characterization of SLE patients

Based on CMR, LVEF was reduced (< 50%) in 2 (3%) patients. Diagnosis and grading of diastolic dysfunction by echocardiography were feasible in 70 patients. Nine patients (13%) had diastolic dysfunction of whom 4 patients had mild (grade 1) and 5 patients had moderate (grade 2) diastolic dysfunction.

### Comparison of SLE patients with healthy controls

Comparison of CMR parameters in SLE patients and healthy controls are presented in Table 2. LVEF was 2 pp higher in SLE patients (p = 0.05); however, both groups were within normal ranges. RV volumes were 6–8% lower in SLE patients compared to healthy controls; however, due to a similar reduction in end-diastolic and systolic RV volume, there was no difference in RV systolic function between SLE patients and healthy controls.

**Table 2:** Left and right ventricular size and function and myocardial T1 mapping by cardiac magnetic resonance in 79 patients with systemic lupus erythematosus and 79 age- and sex-matched healthy controls

CMR parameter	Healthy controls	SLE patients	p-value
Left and right ventricular size			
LVEDVi (ml/m^2^)	78 (70–86)	77 (71–91)	0.51
LVESVi (ml/m^2^)	28 (25–31)	26 (32–32)	0.09
LVSVi (ml/m^2^)	50 (45–56)	54 (47–59)	0.03*
RVEDVi (ml/m^2^)	92 (85–98)	87 (78–95)	0.02*
RVESVi (ml/m^2^)	40 (35–44)	36 (32–41)	0.01*
Left and right ventricular systolic function			
LVEF (%)	65 ± 5	67 ± 7	0.03*
RVEF (%)	57 ± 5	57 ± 9	0.99
Myocardial fibrosis			
T1 (ms)	1019 ± 25	1031 ± 36	0.01*

Values are mean ± standard deviation or median (quartiles)

*CMR* cardiac magnetic resonance, *LVEDVi* Left Ventricular End-Diastolic Volume Index, *LVEF* left ventricular ejection fraction, *LVESVi* Left Ventricular End-systolic Volume Index, *LVSVi* Left Ventricular Stroke Volume Index, *RVEDVi* Right Ventricular End-diastolic Volume Index, *RVEF* right ventricular ejection fraction, *RVESVi* Right Ventricular End-systolic Volume Index

*p ≤ 0.05

T1 mapping was feasible in 77 patients. T1 values were 12 ms higher in SLE patients compared to healthy controls (p = 0.01), as illustrated in Fig. 1. With an upper normal reference of 1068 ms, 14% of SLE patients had elevated T1 values.

### Late gadolinium enhancement and extracellular volume in SLE patients

Gadolinium contrast was administered to 51 out of 79 SLE patients. Of the 28 patients not receiving gadolinium contrast, 7 patients had a low eGFR (≤ 45 ml/min/1.73 m^2^), 3 patients refrained from receiving contrast, 4 patients developed claustrophobia or discomfort before administration, and 14 patients did not receive contrast due to logistic reasons (e.g., gadolinium contrast agent not available or no intravenous access). ECV mapping was feasible in 40 patients.

Mean ECV was 28.1 ± 2.8%. LGE was present in 10/51 (20%) SLE patients who received gadolinium contrast. The pattern of LGE was ischemic in 8/10 patients and non-ischemic in 2/10 patients. The median (interquartile range) LGE relative volume was 7 (4–10) %. There was no clear LGE distribution pattern in ischemic patients; however, the two patients with non-ischemic LGE both presented with septal mid-wall LGE. LAC was present in 67% of patients with LGE. Of patients with LGE, 70% were positive for LAC, and of patients without LGE, 32% were positive for LAC (*χ*^2^ (1, N = 51) = 7.53, p = 0.01). One of the patients presenting with both LGE and LAC was a 35-year-old male with known antiphospholipid syndrome due to cerebral infarcts and deep vein thrombosis but no history of heart disease. He presented on CMR with extensive subendocardial and transmural LGE in all three coronary territories (Fig. 2), a T1 value of 1102 ms, and an LVEF of 35%. Subsequent CT coronary angiogram was normal, and imaging findings were attributed to multiple micro emboli and/or thrombi.

T1 and ECV trended towards being elevated in patients with LGE; however, not statistically significantly. As expected, T1 and ECV values were strongly correlated (r = 0.6, p < 0.001).

### Associations between myocardial fibrosis and ventricular size and function

Increased LV volumes were associated with both increased T1 and ECV values (p < 0.05) (Table 3) when adjusted for age, sex, and traditional risk factors. Further, a lower LVEF was associated with increased T1 values (p < 0.001), and increased left atrial volume index was associated with higher ECV (p = 0.05). LGE was neither associated with LV size nor function besides slightly reduced e’ velocity (p = 0.04). No other significant associations were found between measures of myocardial fibrosis on CMR and biventricular size and function.

**Table 3:** Sex-and age-adjusted linear and logistic regression models of the association between measures of myocardial fibrosis by cardiac magnetic resonance and biventricular size and function in patients with systemic lupus erythematosus

Imaging parameter	T1 (n = 77)			LGE (n = 51)			ECV (n = 40)		
	β	SE	P	β	SE	P	β	SE	p
Left and right ventricular size									
LVEDVi (ml/m^2^)^†^	165.61	54.23	0.003*	11.82	6.85	0.08	11.65	5.53	0.04*
LVESVi (ml/m^2^)^†^	108.27	27.22	< 0.001*	2.87	0.99	0.32	0.71	3.07	0.81
LVSVi (ml/m^2^)^†^	26.95	61.25	0.66	12.62	8.46	0.14	18.22	6.41	0.01*
RVEDVi(ml/m^2^)^†^	6.38	67.81	0.93	− 5.02	7.72	0.52	0.06	0.04	0.14
RVESVi (ml/m^2^)^†^	3.96	46.67	0.93	− 3.53	4.79	0.46	5.42	8.91	0.55
Left and right ventricular systolic function									
LVEF (%)	− 1.94	0.53	< 0.001*	− 0.03	0.05	0.63	0.04	0.06	0.51
RVEF (%)	0.33	0.54	0.54	− 0.03	0.04	0.52	0.07	0.05	0.14
Diastolic function									
E/A ratio^†^	− 19.92	29.98	0.51	− 4.07	2.97	0.17	1.65	3.27	0.62
LAVi (ml/m^2^)^†^	70.21	35.87	0.05	− 2.24	4.15	0.59	9.37	4.48	0.05*
e’ velocity (cm/s)	− 2.00	1.62	0.22	− 0.45	0.22	0.04*	0.06	0.16	0.70
E/e’ ratio^†^	48.17	25.33	0.06	4.77	2.71	0.08	2.11	2.43	0.39
TR velocity (m/s)	− 12.48	10.58	0.24	0.25	1.17	0.83	− 1.04	1.09	0.35

### Associations between myocardial fibrosis and SLE-specific risk factors

The presence of LAC was associated with the presence of LGE (p = 0.04) in the adjusted regression model (Table 4). Further, there was a trend towards higher T1 in LAC positive patients (p = 0.08), as well as a trend towards a negative association between LN and the presence of LGE (p = 0.0521) and a positive association between higher CKD class and LGE (p = 0.0504). No other associations were found between measures of myocardial fibrosis on CMR and SLE-disease characteristics.

**Table 4:** Adjusted multiple linear and logistic regression models of the association between measures of myocardial fibrosis by cardiac magnetic resonance and SLE-specific risk factors in patients with systemic lupus erythematosus

Disease characteristic	T1 (n = 79)			LGE (n = 53)			ECV (n = 40)		
	β	SE	P	β	SE	P	β	SE	p
SLEDAI-2 K^†^	10.24	15.20	0.50	− 4.40	3.55	0.22	− 1.12	2.00	0.58
LN	0.40	9.22	0.97	− 7.10	3.65	0.05	− 0.42	1.19	0.72
CKD	9.62	6.96	0.17	4.93	2.52	0.05	0.51	0.85	0.56
IgM aPL	− 18.89	10.69	0.08	− 3.32	2.20	0.13	− 1.12	1.48	0.46
IgG aPL	− 6.25	10.43	0.08	5.60	2.98	0.06	0.73	1.33	0.59
LAC	16.9	9.54	0.08	3.87	2.20	0.04*	1.55	1.13	0.18

Adjusted for age, sex, hypertension, hypercholesterolemia, and smoking (ever)

Variable definitions are provided in Tables 1, 2 and 3

*p ≤ 0.05

^†^Log-transformed due to non-normality

## Discussion

In this study controlled for traditional cardiovascular risk factors, we found that SLE patients had (1) signs of myocardial fibrosis estimated with T1 mapping compared to healthy controls, and (2) measures of myocardial fibrosis in SLE patients were associated with the presence of LAC. Given that SLE patients are at increased risk of heart failure not only explained by traditional risk factors, it seems important to identify other underlying SLE-related risk factors.

### Prevalence of myocardial fibrosis in SLE patients

Previous studies have reported of LGE in SLE patients with a prevalence ranging from 24 to 61% [10–14, 35, 36]; however, these were all small studies (< 50 SLE patients) with widely different inclusion- and exclusion criteria. Contrary to our study, most have reported of mainly non-ischemic LGE patterns. This discrepancy is likely due to different patient selection and small sample sizes. In one study [12], patients with ischemic LGE were excluded, and patients were mostly younger [11, 12, 14, 35, 36] with shorter disease duration. Further, the analysis of contrast images are reader and image quality dependent, and especially the distinction between non-ischemic mid-wall LGE and artefacts can be challenging if nulling of the myocardium is not perfect. We might have been more conservative in our detection of non-ischemic LGE, which could explain some of the discrepancy with previous studies. Larger studies are however warranted to establish more robust data. Nevertheless, our findings support current evidence that LGE is highly prevalent in SLE patients. Whether LGE is more prevalent in SLE patients compared to healthy controls remains vastly unknown since gadolinium contrast agents are rarely administered to healthy subjects. However, three case-control studies of SLE patients reported of a 0% LGE-prevalence in the control group [12, 36, 37]: Thus substantially lower than the 20% detected in this study cohort.

In the current study, T1 values were elevated in SLE patients compared to healthy controls. Despite a substantial overlap, 14% of SLE patients were above the upper reference value (1068 ms) of the control group. Even though vastly heterogenous, a few studies have similarly found T1 values to be elevated in SLE patients compared to healthy controls [12, 37–39]. In a recent paper by Shalmon et al. [37], SLE patients with suspected myocarditis and antimalarial-induced cardiomyopathy had higher T1 levels compared to controls as well as higher prevalence of LGE. Further, in a retrospective case-series [40] of 11 SLE patients (27% with coronary artery disease, 45% with hypertension, 9% with hyperlipidemia, and 36% with end-stage renal disease) with endomyocardial biopsies, 10 patients had non-specific interstitial fibrosis. T1 mapping is generally considered a technique to quantify diffuse myocardial fibrosis. However, T1 values can be increased in replacement fibrosis as well if T1 images cut through these areas. We did not find T1 nor ECV values to be elevated in patients with LGE compared to patients without LGE despite a tendency for LGE-positive patients to span through a greater range of T1- and ECV-values. This might be either due to (1) a power issue, (2) T1 images not cutting through areas of LGE, which were often small, (3) a difference in underlying pathophysiology causing replacement fibrosis in some patients and diffuse fibrosis in other patients, or that (4) increased T1 values could represent diffuse edema/inflammation and not fibrosis. However, T1 values were not associated with disease activity in our analyses.

### Association between LAC and myocardial fibrosis in SLE patients

Our group has recently described an association between the presence of LAC and diastolic impairment as well as cardiac remodeling during a 5-year follow-up of the present SLE cohort [17]. We speculated whether these changes in cardiac size and function were due to a LAC-mediated development of myocardial fibrosis. In this study, LAC was associated with the presence of LGE, but not T1 nor ECV values. Most LGE-patterns were ischemic despite most patients having no history of coronary artery disease, and thereby possibly represent subclinical small-vessel disease such as thrombosis in distal branches of coronary arteries. This supports the hypothesis of a potential link between LAC and microvascular dysfunction [41] and/or atherosclerosis [42] due to endothelial activation and recruitment of platelets, neutrophils, monocytes, and the complement system [43]. However, a definitive diagnosis of microvascular dysfunction usually relies on invasive coronary angiography and/or non-invasive techniques such as positron emission tomography or CMR with a rest and stress myocardial perfusion study [44]. As these tests were out of the scope of this study, we cannot draw a conclusive link between our LGE-findings and microvascular dysfunction.

To our knowledge, only Mavrogeni et al. [36] have investigated the association between LAC and myocardial fibrosis by CMR in SLE patients. They found no association between the presence of LAC and LGE: however, this was in a group of 44 patients with antiphospholipid syndrome, either primary or SLE-related, and with a high prevalence of LAC (80%) and not adjusted for traditional cardiovascular risk factors.

The fact that LAC was not associated with T1 nor ECV values could be a power issue but could also suggest other mechanisms to be responsible for development of diffuse fibrosis and/or edema. Numerous studies have reported of elevated T1 and ECV values as well as LGE in other diseases characterized by chronic inflammation such as rheumatoid arthritis [45], systemic sclerosis [46], and type 2 diabetes [47]. It thus seems evident that multiple factors besides LAC are involved.

### Association between myocardial fibrosis and ventricular size and function

Given previous findings of impaired diastolic function and increased LV size in SLE patients [16, 17], we investigated whether these parameters were associated with myocardial fibrosis. LV volumes were not significantly increased in SLE patients compared to healthy controls; however, increased volumes as well as reduced LVEF were associated with increased T1 and ECV values in SLE patients. As myocardial fibrosis is highly prevalent in dilated cardiomyopathy and correlates with prognosis [48], it is tempting to draw similar connections in SLE patients.

RV volumes were reduced in SLE patients compared to healthy controls. Few case-control studies have reported of RV-volumes in SLE-patients. A recent case-control study by Pu et al. [49] found no difference in RV-volumes. Our findings on this might be due to healthy controls being more physically active or an actual SLE-related pathophysiological mechanism.

A few measures of diastolic function on echocardiography were associated with LGE and ECV values (e’ velocity and left atrial volume index, respectively), indicating that myocardial fibrosis in SLE patients does somewhat impair the elasticity, compliance, and recoil function of the LV. However, diastolic function is complex, and no single measure can classify diastolic impairment.

Feature-tracking by CMR (FT-CMR), or myocardial strain, quantifies ventricular contractile function and has gained attention in recent years. FT-CMR has the potential to detect subclinical myocardial dysfunction and has shown to have prognostic value [50]. Impaired strain by FT-CMR has previously been correlated with LGE and histology in hypertrophic cardiomyopathy [51] and LGE extend in ST-segment elevation MI [52]. Only a few studies have associated FT-CMR with myocardial fibrosis in SLE. Wu et al. [53] found that elevated ECV in SLE patients was associated with impaired biventricular strain. Puntmann et al. [12] found that T1 was associated with reduced longitudinal strain in SLE patients, but that LGE was not associated with strain. FT-CMR could thus be an indirect measure of myocardial fibrosis in SLE. FT-CMR has the advantage above LGE that it does not require gadolinium contrast, and further the advantage above T1 that no additional images besides standard cine images are required. It could thus serve as an additional marker in patients undergoing CMR, but further studies on this are warranted.

### Clinical implications

LV size and LVEF were not impaired in SLE patients compared to healthy controls. Further, only few had clinically significant heart disease. Most findings including increased T1 values and a high prevalence of LGE thus seem subclinical. However, given that LAC was associated with myocardial fibrosis, a known precursor of heart failure, it seems important to further investigate the prognostic implications of the current findings.

### Limitations

In this study, we defined myocardial fibrosis as the presence of LGE and/or increased T1 and ECV values. However, these methods are not 100% specific and could all represent varying degrees of inflammation/edema. Also, despite ischemic and non-ischemic pattern recognition in LGE imaging, the underlying pathophysiology or condition causing myocardial fibrosis (e.g., infarct, amyloid deposition, edema) remains uncertain without an endomyocardial biopsy. To overcome some of these limitations, we will in future studies implement T2 mapping as well, which is more specific for edema.

We chose not to administer gadolinium contrast to healthy controls in this study due to ethical considerations. Consequently, we were not able to compare the prevalence of LGE and ECV values between SLE patients and healthy controls, which would have strengthened our results.

FT-CMR was not included in this study; however, we are determined to investigate this in our cohort in future studies.

This was a cross-sectional study, and we have no prognostic information regarding the clinical significance of the association between LAC and myocardial fibrosis. However, we hope to investigate this in future follow-up studies.

Finally, as previously described by our group [17], there might be some degree of collinearity in our regression analyses.

## Conclusion

In this cross-sectional case-control study of 79 SLE patients, myocardial fibrosis was more prevalent in SLE patients and associated with the presence of LAC. Findings were mainly subclinical but given the known link between myocardial fibrosis and heart failure, this study raise awareness of LAC as a potential prognostic marker and pathophysiological mediator behind cardiac dysfunction in SLE.
